# Collaborators as a key to survival: an ethnographic study on newly graduated doctors’ collaboration with colleagues

**DOI:** 10.1186/s12909-022-03655-6

**Published:** 2022-08-05

**Authors:** Tine Lass Klitgaard, Diana Stentoft, Nicolaj Johansson, Mette Grønkjær, Susanne Backman Nøhr

**Affiliations:** 1 Department of Postgraduate Medical Education, Aalborg University Hospital, Aalborg, Denmark; 2 Department of Clinical Medicine, Aalborg University, Aalborg, Denmark; 3 Department of Health Science and Technology, Aalborg University, Aalborg, Denmark; 4 Clinical Nursing Research Unit, Aalborg University Hospital, Aalborg, Denmark

**Keywords:** Collaboration, Ethnography, Hospital organisation, Interprofessional collaboration, Newly graduated doctors, Postgraduate medical education

## Abstract

**Background:**

Newly graduated doctors find their first months of practice challenging and overwhelming. As the newly graduated doctors need help to survive this period, collaborators such as peers, senior doctors, registered nurses and other junior doctors are crucial. However, little is known about what characterise these collaborations, and how much is at stake when newly graduated doctors are striving to establish and maintain them. This study aims to describe and explore the collaborations in depth from the newly graduated doctors’ point of view.

**Methods:**

We conducted 135 h of participant observations among newly graduated doctors (*n* = 11), where the doctors were observed throughout their working hours at various times of the day and the week. Furthermore, six semi-structured interviews (four group interviews and two individual) were carried out. The data was analysed thematically.

**Results:**

Newly graduated doctors consulted different collaborators (peers, senior doctors, registered nurses, and other junior doctors) dependent on the challenge at hand, and they used different strategies to get help and secure good relationships with their collaborators: 1) displaying competence; 2) appearing humble; and 3) playing the game. Their use of different strategies shows how they are committed to engage in these collaborations, and how much is at stake.

**Conclusions:**

Newly graduated doctors *rely on building relationships with different collaborators* in order to survive their first months of practice. We argue that the collaboration with peer NGDs and registered nurses has not received the attention it deserves when working with the transition from medical school. We highlight how it is important to focus on these and other collaborators and discuss different work-agendas, mutual expectations, and interdependence. This could be addressed in the introduction period and be one way to ensure a better learning environment and a respectful interprofessional culture.

## Introduction

Despite an increased focus on how to improve the transition from medical school to clinical work as doctors, the period is still perceived as challenging, overwhelming and very stressful [1–9]. In a previous ethnographic study [8], we found that NGDs struggled in their new role because of a lack of local know-how, problems with time management and the feeling of sudden responsibility. To resolve these struggles, NGDs turned to their colleagues. Thus, the collaborators became the NGDs’ salvation.

The term *collaborator* is not an untouched phenomenon in medical education. It is one out of seven key competences in the CanMEDS [10], which is a widely accepted and applied competency framework that describes the abilities doctors require to effectively meet the health care needs of the patients. Here, the role of collaborator is described as essential for *safe, high-quality, patient-centred care*, and it requires *trust, respect […] and pursuing common goals and outcomes* [10]. The literature on collaboration describes how interprofessional collaboration can increase patient safety and enhance health outcomes, and there is a great interest in moving interprofessional education and collaborative practice forward as it can positively contribute to some of the world’s health challenges [11]. The focus is primarily on how to improve collaboration through interprofessional education such as simulation training or workshops [12].

Some studies touch upon the importance of the collaborators during the first months of practice as NGDs, and how good relationships may ease both stress and anxiety [1, 8, 13–15]. However, these studies only briefly describe the importance of collaborative relationships, but not what characterisethem. Bernabao et al. [13] list factors such as effective communication, personality, trust, prior exposure and possessing clear expectations as important to establishing well-functioning collaborations. In our previous study, we found that the NGDs’ collaborative relationships were not always unproblematic: Although patient care was the overriding objective for all staff, different agendas and priorities appeared when demands on patient flow and a high work pace challenged the NGDs [8]. Based on the existing research on the important role of collaborative relationships, there is still a shortage of knowledge on these collaborations from the NGDs’ point of view. Thus, in this study we aim to explore what characterise the NGDs’ collaborations, and which strategies the NGDs use when they are striving to establish and maintain them. These results will provide us with empirical knowledge on the importance of taking the NGDs’ different collaborators into account when planning the transition from medical school.

## Methods

The data used in this study is part of a larger field study focusing on NGDs’ first months of practice. In an already published paper [8], we explored how newly graduated doctors experienced their first months of work in order to understand 1) which struggles they were facing, and 2) which contextual factors within the hospital’s organisation might be essential in this transition. The present paper is a sequel to the former, in which we apply a *selective attention* [16] when re-analysing with a specific focus on the NGDs’ collaborations.

### Study design

We used an ethnographic study design with participant observation and semi-structured interviews. This approach allowed us to obtain a higher level of understanding of the NGDs’ first months of practice. Lived experience is dynamic, and one of the best ways to capture this movement is through engaged ethnographic practice [17–19].

### Study context and participants

The study took place at Aalborg University Hospital in Denmark where approximately 70 NGDs are employed annually. In Denmark, NGDs are required to undergo a foundation Year (FY) before they receive their authorisation to work independently as medical doctors [20]. The NGDs in this study were in the first part of their internship/foundation year programme. Although the first year is part of an educational programme, it is also a fulltime job where the NGDs are expected to contribute to the workforce within the first weeks [20, 21].

The NGDs were all employed at a medical department or at the Accident and Emergency Department (A&E). Besides working in their own departments, all NGDs in this study worked first-line at the A&E where they shared the task of attending to the medical patients and deciding whom to discharge or admit. This work organisation meant that the NGDs often worked remotely from their own departments and had numerous collaborators in many different departments.

Gaining access to the study field involved various steps. First, all involved departments were informed about the study and accepted participation. Second, access had to be planned with the NGDs as their consent to participate was pivotal [18, 22, 23]. As such, access had to be negotiated throughout the entire field study.

### Data generation

#### Participant observation and interviews

The first author donned a white coat and carried out 135 h of participant observation following the NGDs (*n* = 11) around the hospital. The participants were chosen on the basis of availability (who was at work on the particular day) and with variation in gender, medical school, department of employment and prior clinical experience in mind. The observations days were planned on the basis of the work schedule (when NGDs were present), and the arrangements of who to follow was planned before the morning conference. As our approach was explorative, we aimed to participate in as many different situations as possible and since the work flow and time pressure are diverse throughout the day and week we found it important to explore the NGDs’ work at various times. This entailed introduction meetings, conferences, ward rounds and shifts at different days of the week as well as times of the day. Whenever the NGDs interacted with patients or colleagues, the fieldworker remained primarily in the background. All observations (physical spaces, objects, the people present, their activities, the relations and interactions) were recorded and described in detail [23].

In ethnography, interviewing, listening and observing are continuous. However, to get a more thorough understanding of the collaborations from the NGDs’ point of view, the participant observations were supplemented by semi-structured interviews [18, 24–26]. As the study aimed to explore both how the NGDs experienced their first months of practice and how the hospital organisation seemed to influence this, group interviews (*N* = 4, NGDs = 21) became the primary interview method, as in these interviews it is possible to explore various perspectives and different nuances and discover conflicting ideas [18]. For practical reasons, two individual interviews were conducted as well. These were with NGDs who could not participate in the group interviews, but who showed an interest in contributing. All interview recordings were transcribed verbatim.

### Data analysis

Field studies do not proceed in linear model, but cyclically in which the processes of *writing down*, *analysing* and *writing up* are indistinguishably linked [18]. This means that the analysis was not a distinctive phase, but an ongoing procedure. In this study, we examined the data focusing on the NGDs’ collaborations. This meant that all data (field notes and formal interviews) were investigated thematically [27] focusing on the collaboration between the NGDs and their colleagues. Field notes and transcripts were coded regularly in search of themes. The codes were then thematised by identifying common patterns and similarities, e.g. with whom the NGDs collaborated, concerning what and how the NGDs acted in such situations. The first author performed the preliminary coding of data, and all authors contributed to the discussion and interpretation of the findings throughout the analytical process.

### Reflexivity

A central premise of qualitative research is that researchers, as humans studying other human lives, are inevitably and inextricably implicated in what they study [19]. Therefore, it is crucial for the researchers to be explicit about their own role in the research [18]. The first author, who conducted the fieldwork and interviews, is an anthropologist, and the co-authors had different backgrounds and experiences included a medical doctor, a registered nurse, an individual with a PhD in higher education and learning. Several of the authors had experience with higher education and learning processes. The authors’ diverse background provided rich discussions and perspectives on the project and challenged both the methods, data generation, analysis and results.

## Analysis and results

In this study, we explore the collaborations from the NGDs’ point of view. Firstly, we describe *who* their most essential collaborators were and *what* they predominantly were collaborating on. Secondly, we explore *how* the NGDs employed different strategies when striving to establish and maintain their collaborations. In the discussion, we address the *why* to explore what is at stake.

### The collaborators and the nature of collaboration

**Fig. 1:**
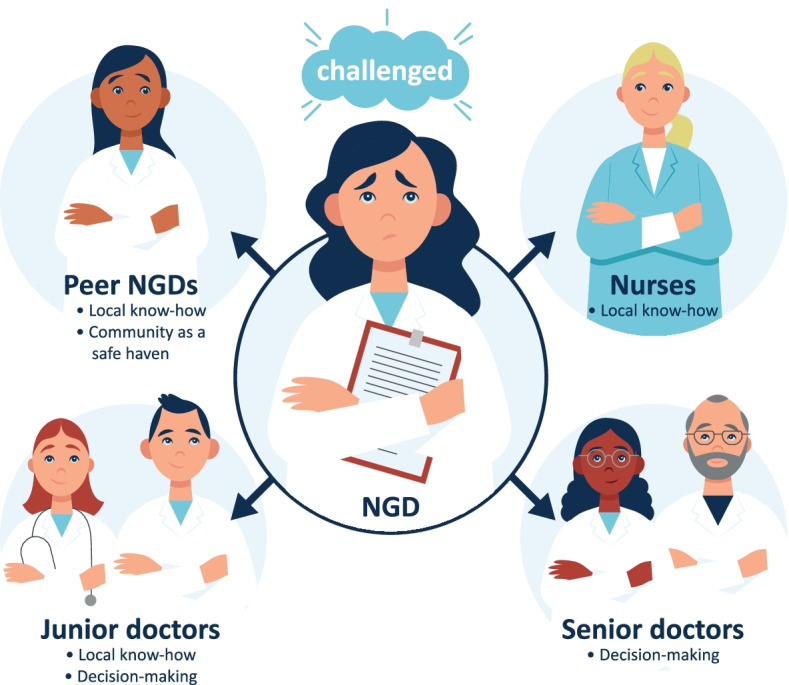
Newly graduated doctors’ collaborators and the reasons they were consulted

During the field study, it became evident that the NGDs consulted different collaborators depending on the challenge they were facing: peer NGDs were seen as a safe haven where uncertainty could be shared; registered nurses were consulted about local know-how; senior doctors were addressed in decision-making; and junior doctors were addressed concerning decision-making and local know-how (see Fig. 1). It is important to notice that the figure illustrates who the NGDs predominantly consulted when in need of help and is as such a simplification. The NGDs sometimes asked registered nurses about clinical decisions, for example which blood test to order, and the NGDs sometimes consulted senior doctors about local know-how, for example where the nearest place to dictate was. However, the aim of the figure is to point out the prevailing and preferred pattern.

In the transition period, the presence of *peers* was crucial. In the interviews, the NGDs expressed how the community with other NGDs provided a feeling of solidarity and a safe haven where both insecurities, difficult experiences, doubts and “stupid” questions were shared (Fig. 1). One NGD expressed: “I honestly don’t know what I would have done without you guys [nearest peers]”. In the field study, it was likewise evident how the NGDs used one another, e.g. when having doubts concerning the patients or when an NGD felt frustrated about her new workplan, she discussed it with one of her peers.

*The registered nurses* (RN) were constantly consulted by the NGDs about local procedures, and a common phrase during the field study was “how do you usually do this?”. This included both practical issues such as how to use a pager, and issues concerning handling the patients. Contrary to the NGDs, the RNs were predominantly affiliated with one department or unit, which endowed them with experience and knowledge about both local procedures and the patient population of the given department:

NGD8: I also think, still, when I’m called to the department during the night when a patient has died, and the relatives are present and want to talk to the doctor (snorts). If it’s a patient who has died of something I can’t even pronounce, and I have to explain “was the patient in pain? Did it go as planned?”. In such moments I just feel SO incompetent. It’s just crap. Luckily, luckily, LUCKILY I’ve only experienced working with great nurses in such situations who were really an essential support. (Group interview)

When the NGDs trotted across the hospital premises multiple times each shift to see patients and work in various sections of the hospital, the RNs were often the ones present, knowing the history and condition of the patients, and thus they became key collaborators to the NGDs.

While the peers and RNs were often addressed concerning struggles about local know-how, the NGDs consulted the *senior doctors* in decision-making issues; for example, concerning diagnostics, further treatments, admission or discharging (Fig. 1). This was evident when following NGD5 during her shift as she received a patient who remained hypotensive despite being administered large amounts of intravenous fluids. In this case, NGD5 was unable to obtain the support from the senior doctor on call because of more acutely ill patients in the A&E. Even though many peers and RNs were present, NGD5 persisted her waiting and wandering. When she contacted the RNs, it was only to ask if they had seen the physician on call. In the end, NGD5 was flushed and seemed really frustrated. When the observer asked if she was feeling insecure about treating the patient alone, she said: “no, I can always call anaesthesia. It’s just because I don’t know what to do to get on with treating the patient”.

The use of senior doctors in decision-making concerning patients was also evident in the observation that they were *chasing the senior doctors*. There were often several NGDs looking for the senior doctors, and despite the presence of several RNs and NGDs, there were often a crowd gathering around the more experienced doctors:

During the supervision there is much disturbance. Many people are present, there are many who talk or make phone calls, and many NGDs are waiting for the senior doctor. Several seem impatient. They are moving uneasily from side to side, taking deep breaths looking at the clock and their notes. (Field note)

The last group of collaborators were the more experienced *junior doctors*. These were often addressed concerning local know-how and procedures, but also in decision-making (Fig. 1). The consulting with junior doctors in decision-making happened particularly with junior doctors from other departments, for example cardiology or gastrointestinal surgery. As junior doctors were more accessible, the NGDs found it easier and contributing to the pace of work to ask junior doctors present rather than contacting seniors on call.

### The NGDs’ strategies in the interactions with collaborators

During the field study it became apparent that the NGDs actively committed themselves to establishing and maintaining good relationships with their collaborators. In this endeavour, they used different strategies: 1) Displaying competence; 2) Appearing humble; and 3) Playing the game. These three strategies were performative and were neither exhaustive nor completely separated.

#### Displaying competence

The NGDs were acutely aware of projecting competence when collaborating with colleagues. This was strongly related to demonstrating independence. Sentences and words used by the NGDs like “not being a burden” and “disturb” indicate how the NGDs did not want to be an encumbrance to their colleagues by interrupting with what might be seen as “banalities”. This sometimes made them consult other peers or junior doctors before asking the senior doctors, for example, when the NGDs were in doubt about ordering a scan or the correct dose of medicine. When the NGDs needed to consult the senior doctors in decision-making it could take many considerations:

NGD17 is on the fence about whether to call the attending doctor. He is seriously in doubt about calling and thereby waking up [the physician on call] to ask what to do with the patient […] I ask if it is because he has had a bad experience previously when waking up his colleague, but that’s not the case. It is ”probably just one’s own professional pride in being able to handle it yourself” that makes him indecisive. (Field notes)

A few hours earlier during the same shift, NGD17 expressed (after talking on the phone with the senior colleague on-call who was headed home) how it was comforting being told that “you can always just give me a call”. What makes NGD17’s many considerations further paradoxical is the formal rule on conferring patients with senior doctors because the NGDs have not yet received their authorisation to work independently. So even though NGD17 did not have any bad experiences with doctors on call, he was told to call, and the formal rule stated that he should call, he was still in doubt whether to call or not.

The concern about displaying competence was also seen in the collaboration with RNs. For example, when NGD10 followed the advice from the physician on call and ordered an extra scan of a patient, even though the RNs expressed how they found this as a waste of time. Hours later when the result came and nothing was wrong with the patient, NGD10’s first response was “now the nurse probably thinks I’m a fool”.

#### Appearing humble

A common strategy among the NGDs was understating their expertise in order to get help. Comments such as “I’m just an NGD” or “this is my first shift; I don’t know ANYTHING” were commonplace. This was especially conspicuous in the collaboration with RNs. The following quote is from observations in the A&E’s break room, where two NGDs and a small group of RNs were present:

NGD2 tells that today she has half a ”training day” before her first night on duty tomorrow. Multiple times she says (very) loudly that she intends to bring cake ”in order to apologise in advance to the RNs” for asking many questions, since she ”doesn’t know anything”. (Field note)

The same understating strategy was evident when NGD18 leaves the A&E after her first shift and tells the RN, who she has been working with all day, that she was sorry that the RN had to be her “babysitter”. When the NGDs in the interviews were asked about this strategy, they explained how they were warned about the RNs in the A&E from the more experienced NGDs:

NGD17: When I started, I was kind of warned about the nurses in the A&E. I mean in general by the other NGDs who had been in our department […] You shouldn’t feel too bad if you meet someone harsh. (Group interview)

These warnings made the NGDs have reservations and they tried to tone down their conduct, which they feared could induce conflicts. During the observations it became clear, that there was sometimes a tense and sneering atmosphere in the A&E. For example when the RNs asked for a “grown-up doctor” when several NGDs were present, or when NGD19 late at night asked the RNs – not the senior doctor – for permission to have his evening meal, and one of them replied: “Hold on… [addressed the other RNs present] What do you think? Do you think he deserves a break?” follow by all of them laughing. Even though the comments might be said with a tongue in cheek, the NGDs sometimes found the A&E RNs tough and making them feel unwelcome. In an interview where the NGDs discussed how they were struggling when everything was new, NGD16 explained:

Then it helps a lot to call and say (makes the voice “small/innocuous”, pulls up the shoulders): “I’m just new here, so I don’t know”, ‘cause then it’s very hard to yell at people. (Group interview)

This quote illustrates how the NGDs’ used a submissive approach as a strategy to get help: Who could make oneself scold/turn away somebody that new and humble?

#### Playing the game

The NGDs quickly learned that when working first-line, an important task was to free the beds and sustain the flow of patients. The long line of waiting patients put pressure on the staff, and focus was on “turfing” the patients (i.e. moving them from one department to another). First and foremost, this was about the patients’ needs and safety as well as ensuring capacity for new admissions. However, during the fieldwork, it was clear how being someone who contributed to sustaining the flow was highly valued:

I hear how a nurse tells “how it is so nice, when a doctor finally comes who can move things along”. (Field notes)

The quote indicates how contributing to the flow was not only about patient care but also a matter of being well-reputed among colleagues. In the interviews, the NGDs discussed how this sometimes meant bypassing the reflections and thereby potential learning situations. Instead of doing all the reflections and investigations themselves, they sought answers from their colleagues to get the patients through faster.

NGD6: ”It’s often a productivity demand, and it impedes all sort of opportunities for education, in my view. That’s my opinion (the others laugh). It’s like: *well, that doesn’t matter, now I just have to get going, and it’s probably good enough, right?*”. (Group interview)

The awareness of contributing to the team goal also had an impact on the NGDs’ asking for feedback on their work. A well-known structured observation tool, “Mini-CEX”, was often described as a way to get specific feedback on their work from another professional that observe them with patients. This model however required a more experienced doctor to prioritise this feedback ahead of attending to patients, and it therefore made hard for the NGDs to ask for:

NGD7: I think you should ask for it [mini-cex, observations and feedback]. […] They’re [senior doctors] just so busy […], and they have plenty to do with taking care of their own patients, so you can’t get one of them to go along for the entire round and say “please observe my work…”. I believe that in general it’s all been a bit too pressed for time for me to think that this is something I could to do. (Group interview)

The quote illustrates how the NGDs were often opting out the opportunity for feedback (and thus a possible learning situation) because they did not want to interrupt the senior doctors by asking many questions or requesting feedback. However, receiving feedback is an important part of being a trainee doctor in order to learn and demonstrate mandatory competences.

## Discussion

In this study, we describe and explore the NGDs’ collaboration with peers, RNs, junior and senior doctors from the NGDs’ point of view. Our analysis demonstrates that the NGDs do not just call a random colleague when in need of help but choose their collaborator dependent on the challenge at hand. Furthermore, we found that the NGDs used different strategies such as *displaying competence, being humble*, and *playing the game* when establishing and maintaining the collaborations. The use of strategies shows how important collaborations are in accomplishing day to day work, and how much is at stake to the NGDs.

To explain the NGDs’ use of strategies, it is relevant to include Goffman’s concept of *impression management* [28] to explain the motivations behind human performances in everyday social interactions. Goffman [28] uses a theatrical vocabulary when analysing how individuals steer interactions: Individuals are “performers” who try to convey a certain impression to their “audiences”. The theory hence presumes that people have a certain degree of control over the way they are perceived by their audience. As newcomers, the NGDs are dependent on their collaborators and this puts them in a position that demands many considerations in relation to establishing and maintaining these collaborations. We found that the NGDs invested themselves (time and energy) in inferring the behavior that they expected would be most effective when they needed help from their collaborators. In some situations, they are absorbed with *displaying competence* and not being an encumbrance to their colleagues. In other situations, they use the opposite strategy by *appearing humble* to reduce the risk of conflicts and legitimate their need for help. Neither of these shifting performances seems to be in accordance with their inner feeling of competence and capability [8]. This need for alternating strategies and performances puts additional stress on top of the already known challenges of being an NGD (e.g. acutely ill patients, the feeling of sudden responsibility, decision making, and lack of local know-how). Also, the strategy of *playing the game* needs attention, because, although it is an important learning object to contribute to the workflow at the hospital, it meant neglecting potential learning situations (e.g. asking for feedback) and/or not tending to one’s own needs (e.g. breaks, food or drinks).

The importance of collaborators in the professional development of the NGDs has been noted by other researchers. For many years, attention within medical education has been directed at students’ and residents’ professional identity formation, which is defined as a dynamic process achieved through socialisation [29, 30]. Cruess et al. emphasise *role models, mentors and the accumulation of individual experiences* as the most powerful factors influencing the shaping of a professional identity [29]. Such role models and mentors are most frequently synonymous with doctors. This emphasis might at first seem rational as the NGDs strive to fit the white coat – become *doctors* – and thus their role models (i.e. “individuals admired for their ways of being and acting as professionals”) are other doctors [29, 31]. The importance of the senior doctors’ presence for the NGDs’ development is also pointed out, both in the literature [32] and in our fieldnotes, where the NGDs were very preoccupied with the opportunity for back-up and supervision during their introduction period.

However, with this one-sided focus on other doctors as role models and mentors, there is a risk of neglecting the role of other collaborators. In our study, the collaboration with the RNs is a significant and important addition, but this is not to our knowledge specifically explored in the existing medical education literature on collaborations. Brennan et al. describe how the lack of support from senior doctors made the NGDs aware of how much they could ask RNs and other doctors about [1], and Bernabao et al. found that NGDs often strive to gain the respect of the RNs as they possess the greatest knowledge of the patients and the system [13]. This is in line with our results as RNs were the ones addressed when the NGDs struggled with local know-how, and often the RNs were the only ones present. However, we observed how conflicting agendas and a harsh tone of voice in communication could challenge the collaborations between RNs and NGDs.

Cruess et al. [29] argue that how NGDs are treated by their relations (e.g. other healthcare professionals) has a significant impact on their sense of self. One might wonder why the crucial collaboration with the RNs has not received more attention previously; both in the literature, but also as a part of the under- and postgraduate medical education. A better interprofessional collaboration should not only focus on increasing patient safety and enhancing patient care, but also on the interdependence in the collaboration. The RNs wanted the patients to be ready for either discharge or admittance to another department as fast as possible, and in order to do this, they are dependent on the NGDs. At the same time, the NGDs are dependent on the RNs’ local knowledge and experience with the patients. Thus, a focus area could be on how to improve the working environment with emphasis on both the importance of a respectful interprofessional culture, the RNs’ role (sometimes as masters of maintaining patient flow) and the NGDs as newcomers. This could be addressed when planning undergraduate and postgraduate medical education, e.g. through introduction to each other’s work and/or creating opportunities to align expectations.

Another important collaborator is the peer NGD. In our study, we found how peers were crucial to the NGDs in the transition period, and how the community of peers were a safe haven where insecurities, difficult experiences, doubts and “stupid” questions were shared and legitimate. This is in line with the findings of Bernabeo et. al. [13] where the NGDs prioritised their relationships with (experienced) peers as it made “things go smoothly”, and Sturman et al. [6] describe how sharing experiences with peers is valuable. What needs to be addressed is how to secure these moments of “sharing”. The concept of peer coaching has developed in medical education [7], but our study shows how the NGDs (also) need peers *in the moment*. The meetings do not only need to be formalised – but natural opportunities for interchanges should also be prioritised and ensured.

### Limitations

In ethnographic fieldwork, people do not necessarily act naturally to a passive observer [33], and thus it is important to have in mind that having an observer present could influence the interactions of the involved doctors and staff.

Our study was conducted in a selected number of departments, in a single hospital, and in a Danish context. The conditions on which we have made our observations may not be similar in other departments, hospitals, or countries, and thus transferability may be limited. Nevertheless, we believe that our descriptions of the collaborations from the NGDs’ point of view could allow others to address similar issues in their own institutions.

## Conclusion

In our study, we found that NGDs rely on building relationships with different collaborators in order to survive their first months of practice. It is not enough to secure back-up and supervision from senior doctors. Our findings highlight the need to pay attention to the NGDs’ access to peers as this provides a feeling of solidarity and a safe haven when their first months of practice become overwhelming. Furthermore, the NGDs need help with local procedures from the RNs and we found that the establishment of these collaborations is not without costs as the NGDs are navigating different performances to meet what they think will be the most advantageous strategy in establishing these collaborations. Thus, it is necessary to rethink the way the NGDs are introduced to their work and learning as new doctors. Including an emphasis on the importance of different collaborators, the opportunity to meet future collaborators and discuss different work agendas and mutual expectations. This could be one way to ensure a respectful interprofessional culture and a better learning environment.

## Data Availability

The data generated and analysed during the current study are available from the corresponding author on reasonable request. The reason why the data is not uploaded along with the manuscript is that the many pages of written fieldnotes and interview transcripts are in Danish and the majority of it is not anonymised.

## Abbreviations

NGD: Newly graduated doctor
A&E: The Accident and Emergency Department
RN: Registered nurse
